# Study on the Antioxidant Effect of Tanshinone IIA on Diabetic Retinopathy and Its Mechanism Based on Integrated Pharmacology

**DOI:** 10.1155/2022/9990937

**Published:** 2022-11-17

**Authors:** Xiaomei Zeng, Ying Deng, Mengxia Yuan, Qi He, Yonghe Wu, Shibing Li

**Affiliations:** ^1^People's Hospital of Ningxiang City, Ningxiang, China; ^2^Hunan University of Chinese Medicine, Changsha, China

## Abstract

**Aim:**

To explore the effect of tanshinone IIA on diabetic retinopathy (DR) and its mechanism.

**Methods:**

GeneCards and OMM databases were used to mine DR-related genes. The chemical structure of tanshinone IIA was searched by PubChem, and the potential target was predicted by PharmMapper. Cystape 3.8.2 was used to visualize and analyze the tanshinone IIA-DR protein interaction network. DAVID ver 6.8 data were used to perform enrichment analysis of the tanshinone IIA-DR protein interaction network. Then animal experiments were carried out to further explore the mechanism of tanshinone IIA in the treatment of DR. Male SD rats were intraperitoneally injected with streptozotocin to establish a diabetes model and were randomly divided into a model group, a low-dose tanshinone IIA group and a high-dose group. Normal rats served as the control group. Hematoxylin-eosin (HE) staining was used to observe the structural changes of the retina; the SOD, GSH-Px, and MDA levels in the retina were detected by the xanthine oxidase method; the expression of VEGF, IL-1*β*, IL-6, TNF-*α,* and caspase-3 mRNA were detected by qRT-PCR; and the Bcl-2, Bax, and VEGFA proteins were determined by the western blot.

**Results:**

A total of 213 tanshinone IIA potential targets and 223 DR-related genes were obtained. The enrichment analysis showed that tanshinone IIA may regulate hypoxia, oxidative stress, positive regulation of ERK1 and ERK2 cascade, steroid hormone-mediated signaling pathway, inflammatory response, angiogenesis, VEGF signaling pathway, apoptosis, PI3K-Akt signaling pathway, TNF signaling pathway, and biological processes and signaling pathways. The structure of the retina in the normal control group was clear, the retina in the model group was not clear, the nerve fiber layer was edema, the retinal cell layers of the tanshinone IIA low-dose group are arranged neatly, the inner and outer nuclear layers are slightly disordered, and the tanshinone IIA low-dose group was large. The structure of the mouse retina was further improved compared with the low-dose tanshinone IIA group. Compared with the model group, the retinal tissue SOD and GSH-PX of rats in the tanshinone IIA group increased, and the MDA level decreased (*P* < 0.05). Compared with the model group, the expression of VEGF, IL-1*β*, IL-6, TNF-*α*, and caspase-3 mRNA in the retina of tanshinone IIA groups was significantly reduced (*P* < 0.01). Compared with the model group, the Bcl-2 protein in the tanshinone IIA groups increased, while the Bax and VEGFA proteins decreased (*P* < 0.05).

**Conclusion:**

Tanshinone IIA may improve the morphological performance of the retina of diabetic rats and inhibit DR, the mechanism of which may be anti-inflammatory, antiangiogenesis, etc.

## 1. Introduction

Type 2 diabetes mellitus (T2DM) refers to a relative lack of insulin. Patients mainly suffer from increased blood sugar due to insufficient insulin secretion or tolerance, as well as polydipsia, polyuria, polyphagia, and weight loss. T2DM is currently one of the most common chronic deficiency disorders in clinical practice. With the aging of the Chinese population and changes in lifestyle, the incidence rate is increasing year by year [1, 2]. The prevalence of diabetes has soared from 0.67% in 1980 to 10.4% in 2013. Diabetes in China is mainly T2DM, and type 1 diabetes and other types of diabetes are rare [3, 4]. Various chronic complications of diabetes are the most important cause of disability and death in diabetic patients. Among the chronic complications of diabetes, diabetic retinopathy (DR) has the highest disability rate, but there is a lack of noninvasive screening methods in clinical practice [5]. Diabetic retinopathy is a common ocular complication in diabetic patients. Its pathological process of retinopathy mainly includes macular edema, vitreous hemorrhage, and retina neovascular glaucoma. DR seriously affects the eyesight of patients and is a common cause of blindness in people of working age. The prevalence of DR in the diabetic population in our country is 25.0%–43.1%, and its incidence is the result of the combined effect of environmental factors and genetic factors. Hyperglycemia, hypertension, and long-term diabetes are important environmental factors that promote diabetes and retina disease [6, 7].

Current studies have shown that long-term hyperglycemia leads to the increase of protein kinase C, glycosylated hemoglobin, and polyol metabolites, which affect the physiological functions of the retina and causes oxidative stress damage [8–10]. Both the diabetic rat models cultured with high glucose and the human retina pigment epithelium showed a significant increase in retinal lipid peroxidation and a decrease in antioxidant enzymes. Studies have shown that antioxidation and anti-inflammatory effects can reduce retinal damage in diabetic rats [11, 12]. Tanshinone IIA is separated and purified from the Chinese herbal medicine Danshen. Studies have shown that tanshinone IIA sodium sulfonate has anti-inflammatory, antioxidant, and vasodilator effects [13], which may decrease nitric oxide (NO), interleukin (IL)-1*β*, IL-6, and tumor necrosis factor (TNF)-*α* [14, 15]. Current research shows that it can interfere with diabetic microvascular diseases [16], especially diabetic retinopathy [17]. But the current mechanism of tanshinone IIA intervention in DR still needs to be further elucidated, especially its molecular network of intervention in DR.

Systematic pharmacology is based on high-throughput omics data analysis, computer simulation calculations, and network database retrieval, to construct and analyze biological networks and then to study the mechanism of drug action and discover innovative drugs [18, 19]. This method starts from the holistic and systematic nature of the interaction among drugs, targets, and diseases and uses a complex network model to express and analyze the pharmacological properties of the research object [20, 21]. It is especially suitable for studying the relationship of multitarget drugs, which is conducive to revealing the complex mechanism of multitarget drugs intervening diseases at the system network level [22, 23]. Therefore, this study will explore the mechanism of tanshinone IIA intervention in diabetic retinal diseases based on systemic pharmacology methods to explore the diabetic retina animal model through target identification, kyoto encyclopedia of genes and genomes (KEGG) pathway analysis, and network construction.

## 2. Materials and Methods

### 2.1. Tanshinone IIA Target Prediction and DR-Related Gene Collection

Tanshinone IIA was retrieved from PubChem (https://pubchem.ncbi.nlm.nih.gov/), the 3D molecular structure of the molecule was downloaded and saved in the “sdf” format. The “sdf” file of tanshinone IIA were input into PharmMapper (https://lilab-ecust.cn/pharmmapper/) for potential target prediction [24]. “Diabetic retinopathy” was used as a keyword to search for DR-related genes in GeneCards (https://www.genecards.org/) [25] and the Online Mendelian Institute of Humans (OMIM) (https://omim.org/) [26]. UniProt (https://www.uniprot.org/) was used to convert potential targets of tanshinone IIA and DR-related genes into official gene symbols (Table S1).

### 2.2. Construction and Analysis of the Protein-Protein Interaction (PPI) Network

The potential targets of tanshinone IIA and DR-related genes were uploaded to the STRING online database with a confidence score >0.4, and the corresponding PPI data were obtained [27]. Then, they were imported into Cytoscape 3.8.2 for network construction. A degree is used as a standard to measure the importance of nodes in the PPI network. The Database for Annotation, Visualization, and Integrated Discovery [28] (DAVID, https://david.ncifcrf.gov/, ver. 6.8) was used to perform target gene ontology (GO) enrichment analysis and KEGG pathway enrichment analysis for genes in the PPI network.

### 2.3. Experimental Materials

#### 2.3.1. Instruments and Reagents

Tanshinone IIA sodium sulfonate (Shanghai No. 1 Biochemical Pharmaceutical Co., Ltd., National Medicine Standard H31022558), 4% paraformaldehyde and 2.5% glutaraldehyde (Beijing Solarbio Life Science and Technology Co., Ltd.), 1% bone microacid (Shanghai Harin Biological Technology Co., Ltd.), lead citrate and 2% uranyl acetate (Shanghai Rongchuang Biotechnology Co., Ltd.), TRIzol (Invitrogen, USA), and VEGF, IL-1*β*, IL-6, TNF-*α*, caspase-3, and *β*-actin upstream and downstream primers (Shanghai Shenggong Company) were used. Tumor necrosis factor-*α* (TNF-a) kit (Lot Number: 20180614), interleukin-6 (IL-6) kit (Lot Number: 20180614), and interleukin-1 (IL-1) kit (Lot Number: 20180614) were purchased from the Nanjing Jiancheng Institute of Biological Engineering. Operating microscope (German Leica, model: M525F40). Inverted biological microscope (Shanghai Optical Instrument Factory, model: 37X F), electrophoresis instrument (Beijing Liu Yi Biological Technology Co., Ltd. Model: DYCZ-40D), hematoxylin-eosin staining (Wuhan Boster Biological Engineering Co., Ltd.), BCA protein detection kit, lysate, PVDF membrane and chemiluminescence reagents, anti-VEGFA antibody (ab52917) (abcam company), anti-Bcl-2 antibody (ab194583), and anti-Bax antibody (ab32503) antibodies (Shanghai Beiyo Time Biotechnology), goat anti-rabbit secondary antibody (American LICOR company), ABI 7500 real-time fluorescent quantitative PCR, MultiskanTM FC full-automatic microplate reader, and iBrightTM CL1500 imaging system (China Thermo Fisher Scientific Technology Co., Ltd.), Allegra X-64R High Speed Table Freeze Centrifuge (Beckman Coulter, USA) were used.

#### 2.3.2. Experimental Animal

Seventy healthy SPF grade male SD rats, weight: 220 ± 10 g, 6-7 weeks old, were purchased from Dongguan Songshan Lake Mingzhu Experimental Animal Technology Co., Ltd., license number SCXK (Guangdong) 2017-0004. The rats were raised in the Central Laboratory of Hunan University of Chinese Medicine, at a temperature of (22 ± 2)°C, and a humidity of 70% ± 5%. Animal experiments have been approved by the Animal Ethics Committee of Hunan University of Chinese Medicine and performed in accordance with the guidelines for the care and use of experimental animals.

### 2.4. Experimental Methods

#### 2.4.1. Animal Modeling

High-sugar and high-fat feed and intraperitoneal injection of streptozotocin (STZ) were used to establish a rat diabetes model. Seventy SD rats were randomly divided into a control group (10) and an experimental group (60). The rats in the experimental group were fed with high-fat diet (10% lard; 20% sucrose; 1.0% bile acid; 2.5% cholesterol; 66.5% standard diet). The rats in the control group were fed with regular feed. After 1 week of feeding, the experimental group was intraperitoneally injected with 30 mg/kg of STZ, and the control group was intraperitoneally injected with the same dose of solvent. The injection was continued for 1 week to construct a hyperglycemia model [29]. The experimental group continued to feed on a high-fat and high-sugar diet for 1 month, until the rats developed hyperglycemia and insulin resistance, and the T2DM model was considered successful. After the T2DM model was successfully constructed, the experimental group continued to feed on a high-fat and high-sugar diet for 1 month until retinopathy appeared, then the DR model was constructed successfully.

Model evaluation: hyperglycemia model: fasting blood glucose (FBG) ≥ 11. 1 mmol/L is considered a successful model construction. T2DM model: the FBG and fasting insulin (FINS) were measured after fasting for one night in rats, and the glucose tolerance test was performed; the FBG ≥ 11.1 mmol/L and the appearance of insulin resistance were successfully constructed [10]. DR model: 4 rats were randomly selected, and the retinas of both eyes were taken for HE staining; the model was constructed successfully if the retina of the rats showed related disease symptoms.

#### 2.4.2. Animal Grouping and Intervention

A total of 70 male SD rats were selected for this experiment, 10 of which were set as the control group, and they were given a standard diet. The same amount of citrate buffer was injected intraperitoneally every day. There were 47 animals left after the model was successfully created. Among them, 17 rats were divided into model groups; they were given a high-fat and high-sugar diet, and the same amount of citrate buffer was injected intraperitoneally every day. 15 rats were present in the tanshinone IIA low-dose group and 15 rats in the high-dose group of tanshinone IIA; they were given a high-fat and high-sugar diet and were injected tanshinone IIA sodium sulfonate 10 mg/kg and 20 mg/kg intraperitoneally each day, respectively. The intervention experiment was conducted for 2 months.

#### 2.4.3. General Condition of Rats

The weight of the rats is measured daily to observe whether there is death. After blood sampling from the tail vein, triglyceride (TG), total cholesterol (TC), high-density lipoprotein cholesterol (HDL-C), low-density lipoprotein cholesterol (LDL-C), and blood sugar levels were measured.

#### 2.4.4. Observation and Selection of Diseased Retina

After the last intervention, the rats were anesthetized with 3% sodium pentobarbital (40 mg/kg). The right eyeball was aseptically removed under a stereo microscope, and the retinal tissue was separated. HE staining: fixed with 4% paraformaldehyde for 24 h and stained with hematoxylin for 5–10 min. After rinsing with distilled water, staining with eosin for 5–10 min, the slide was rinsed again with distilled water, dehydrated with gradient ethanol solution, and then the pathological changes of the retina were observed.

#### 2.4.5. Retina Tissue SOD, GSH-Px, and MDA Level Detection

Each group of retinal tissue was ground into tissue homogenate and centrifuged at low speed (2000 r/min), and then the supernatant was taken. The content of MDA in retinal tissue was detected by the thiobarbituric acid method; the content of SOD and GSH-Px in retinal tissue was detected by the microplate method. The content of MDA, SOD, and GSH-Px per mg of total protein is calculated.

#### 2.4.6. Serum TNF-*α*, IL-1*β*, and IL-6 Level Detection

After blood was collected from the abdominal aorta of the rat, the blood was centrifuged at 4000 r/min at 4°C for 15 min to obtain serum. Serum levels of TNF-*α*, IL-1*β*, and IL-6 were detected with a rat-specific ELISA kit. The operation steps were strictly in accordance with the kit instructions, and the absorbance at 450 nm was detected using a microplate reader.

#### 2.4.7. Expression of VEGF, IL-1*β*, IL-6, TNF-*α,* and Caspase-3 mRNA in Retinal Tissue Detected by RT-qPCR Method

The total RNA from retinal tissue was extracted by the TRIzol method. After the concentration was detected by a microplate reader, the total RNA was reverse-transcribed to obtain cDNA. The SYBR Green I real-time fluorescent quantitative PCR method was used to detect the expression levels of VEGF, IL-1*β*, IL-6, TNF-*α,* and caspase-3 genes. Reaction conditions: 95°C predenaturation for 5 minutes; 95°C denaturation for 30 seconds, 60°C annealing for 30 seconds, 72°C extension for 30 seconds, a total of 40 cycles, and finally, 72°C extension for 10 minutes. The test was repeated 3 times for each sample. The relative expression of each group of genes is calculated according to the formula (2^−ΔΔ^Ct method) (Table 1).

#### 2.4.8. Expression of Bcl-2, Bax, and VEGFA Protein in Retinal Tissue Detected by the Western Blot

The retinal tissue was lysed by adding 400 *μ*l of RIPA lysis buffer containing protease inhibitors on ice, grinding for 30 min to extract total protein, and centrifuging at 10,000 r/min for 10 min to take the supernatant. The protein content was determined by the BCA method. A sample of 30 *μ*g protein was added, and the protein was separated by a 10% SDS-PAGE gel and then transferred to a polyvinylidene fluoride membrane. After the membrane is incubated with the primary antibody and the secondary antibody, the ECL chemiluminescent liquid was added dropwise to develop color, the gel imaging system was used to take pictures, and the Image Pro-Plus analysis system was used to analyze the protein bands.

### 2.5. Statistical Analysis

SPSS 20.0 statistical software was used to analyze the data. The experimental data are expressed as (*x* ± *s*), the data comparison between the two groups uses *t*-test, and the data comparison between the groups uses one-way analysis of variance. *P* < 0.05 indicates that the difference is statistically significant.

## 3. Results and Discussion

### 3.1. Tanshinone IIA-DR PPI Network Analysis

**Figure 1:**
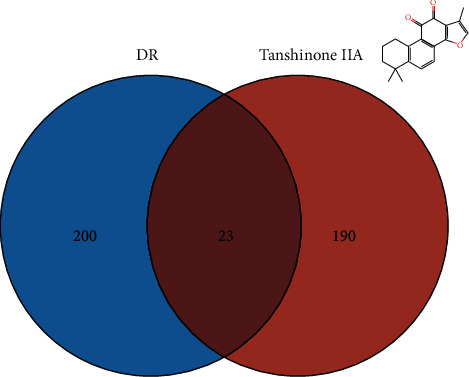
Venn diagram of tanshinone IIA potential target set and DR gene set.

**Figure 2:**
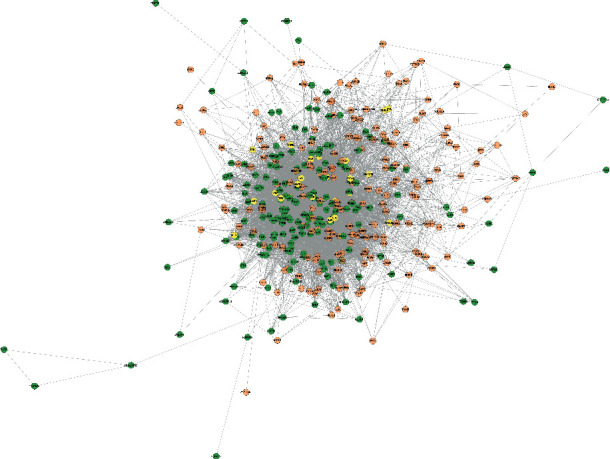
Tanshinone IIA-DR PPI network (green, orange, and yellow circles stand for tanshinone IIA targets, DR genes, tanshinone IIA target-DR target, respectively).

**Figure 3:**
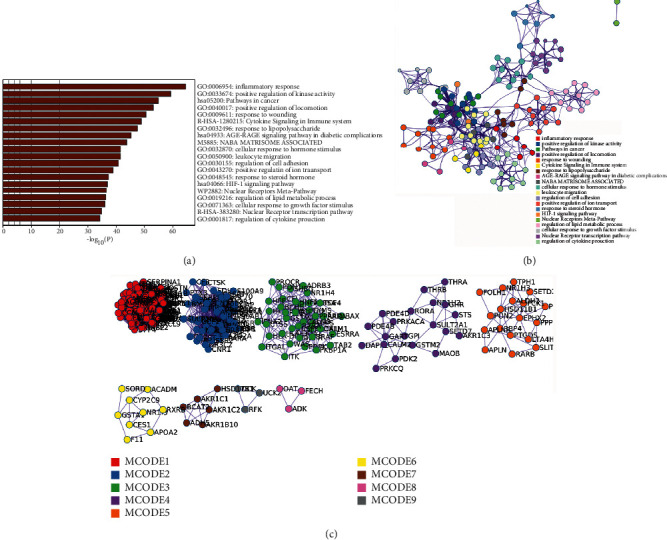
Metascape analysis results. (a) The top enrichment analysis results; (b) PPI network colored by the cluster; (c) the cluster of tanshinone IIA-DR PPI network.

A total of 213 tanshinone IIA potential targets and 223 DR-related genes were obtained. There is some overlap between the tanshinone IIA potential target set and the DR gene set (Figure 1). The interaction among tanshinone IIA potential targets and DR genes was visualized by Cytoscape 3.8.2 (Figure 2). Tanshinone IIA-DR PPI network was composed of 187 tanshinone IIA target nodes, 176 DR gene nodes, 23 tanshinone IIA-DR target nodes, and 7814 edges. Targets that cannot interact are removed from this network. The top 10 target in each target set is (1) tanshinone IIA target set: EGFR (151 edges), MAPK1 (149 edges), SRC (143 edges), CASP3 (136 edges), ESR1 (116 edges), MAPK14 (106 edges), HSP90AA1 (99 edges), IL2 (96 edges), BCL2L1 (87 edges), and F2 (85 edges); (2) DR gene set: INS (226 edges), IL6 (202 edges), VEGFA (190 edges), TNF (186 edges), FN1 (164 edges), EGF (163 edges), MMP9 (154 edges), CXCL8 (144 edges), STAT3 (142 edges), and IL1B (133 edges); and (3) tanshinone IIA-DR target set: ALB (211 edges), IGF1 (145 edges), MAPK8 (136 edges), NOS3 (115 edges), MMP2 (114 edges), KDR (102 edges), PPARG (86 edges), AR (75 edges), REN (71 edges), and SOD2 (67 edges). Metascape was used for preliminary analysis of the tanshinone IIA-DR PPI network (Figure 3).

### 3.2. Enrichment Analysis of Tanshinone IIA-DR PPI Network

**Figure 4:**
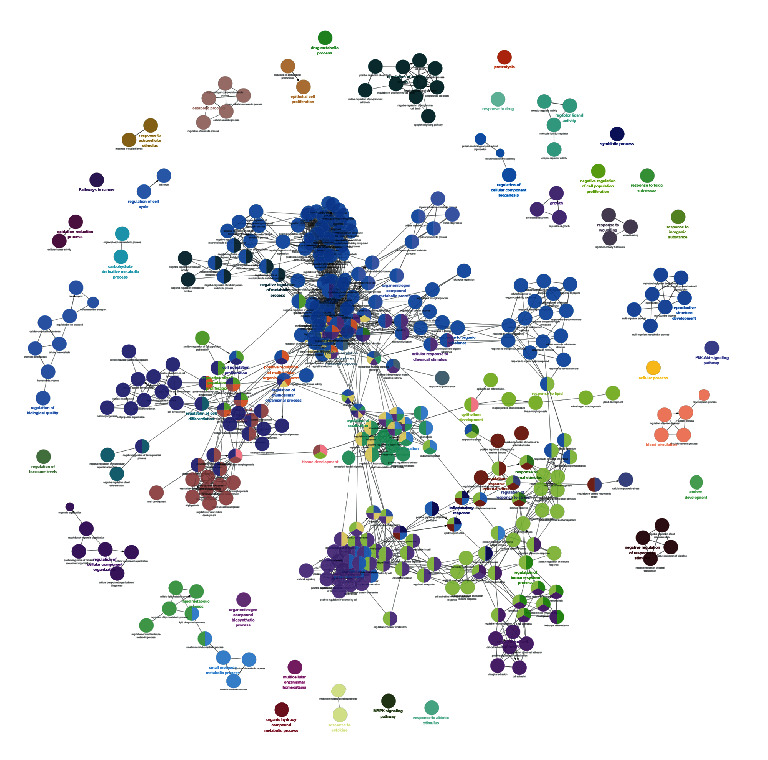
The enrichment analysis of the tanshinone IIA-DR PPI network.

**Figure 5:**
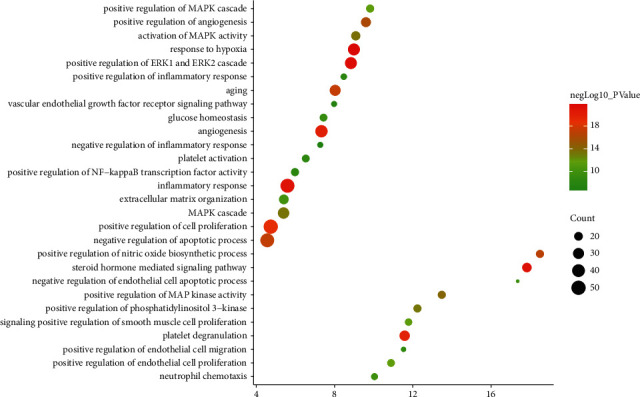
Bubble chart of the BP (*X*-axis stands for fold enrichment).

**Figure 6:**
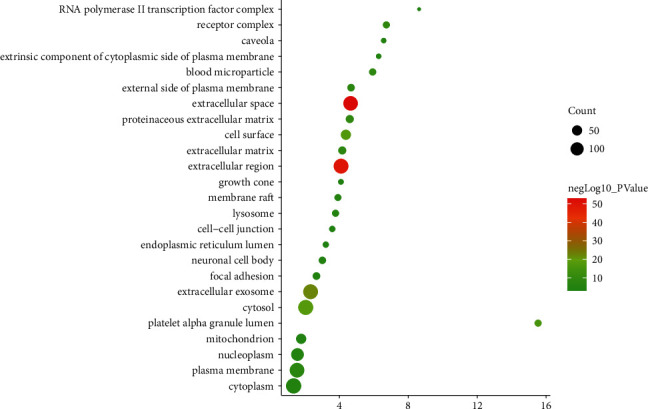
Bubble chart of the CC (*X*-axis stands for fold enrichment).

**Figure 7:**
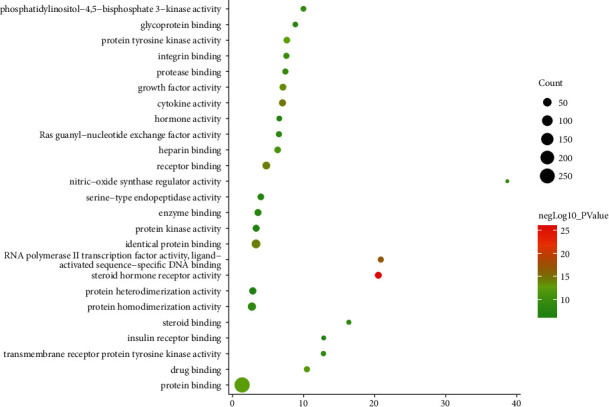
Bubble chart of the MF (*X*-axis stands for fold enrichment).

**Figure 8:**
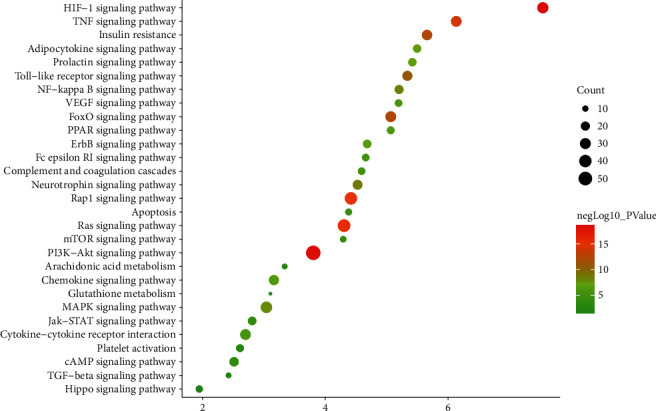
Bubble chart of the signaling pathway (*X*-axis stands for fold enrichment).

The targets and genes in the tanshinone IIA-DR PPI network were input into DAVID for enrichment analysis. The overall enrichment results were visualized by CLUEGO (a plug-in in Cytoscape) (Figure 4). The GO enrichment results include the biological process (BP), cell component (CC), and molecular function (MF). The BP includes response to hypoxia, positive regulation of ERK1 and ERK2 cascade, steroid hormone mediated signaling pathway, inflammatory response, angiogenesis, platelet degranulation, positive regulation of cell proliferation, aging, negative regulation of apoptotic process, positive regulation of nitric oxide biosynthetic process, positive regulation of angiogenesis, positive regulation of MAP kinase activity, activation of MAPK activity, MAPK cascade, positive regulation of phosphatidylinositol 3-kinase signaling, positive regulation of endothelial cell proliferation, positive regulation of MAPK cascade, positive regulation of smooth muscle cell proliferation, extracellular matrix organization, and so on (Figure 5). The CC includes extracellular space, extracellular region, extracellular exosome, cytosol, cell surface, platelet alpha granule lumen, proteinaceous extracellular matrix, receptor complex, blood microparticle, extracellular matrix, external side of plasma membrane, plasma membrane, and so on (Figure 6). The MF includes steroid hormone receptor activity, RNA polymerase II transcription factor activity, ligand-activated sequence-specific DNA binding, cytokine activity, receptor binding, identical protein binding, growth factor activity, protein tyrosine kinase activity, protein binding, drug binding, heparin binding, integrin binding, protease binding, phosphatidylinositol-4,5-bisphosphate 3-kinase activity, protein homodimerization activity, and so on (Figure 7). The signaling pathway includes the VEGF signaling pathway, apoptosis, Fc epsilon RI signaling pathway, HIF-1 signaling pathway, PI3K-Akt signaling pathway, Ras signaling pathway, Rap1 signaling pathway, TNF signaling pathway, FoxO signaling pathway, insulin resistance, Toll-like receptor signaling pathway, neurotrophin signaling pathway, NF-kappa B signaling pathway, MAPK signaling pathway, adipocytokine signaling pathway,and prolactin signaling pathway (Figure 8). The details are shown in Table S2.

In this study, based on the idea of multitarget-multipathway, the integrated pharmacological technology was used to analyze the targets of tanshinone IIA in the treatment of DR, and the molecular mechanism of tanshinone IIA in the treatment of DR was explained, providing a basis for the experimental research and clinical application of tanshinone IIA. As a class of flavonoids, tanshinone IIA has a wide range of anti-inflammatory, antibacterial, and antioxidant activities [13]. Studies have shown that tanshinone IIA has a protective effect on both *in vivo* and *in vitro* retinal pigment epithelial cells induced by oxidation [30]. Tanshinone IIA can significantly inhibit the *in vitro* biological effects of human retinal vascular endothelial cell proliferation, migration, and angioplasty under high glucose environment and can play a certain role in preventing and treating the occurrence of DR and delaying its pathological process [31]. Through tanshinone IIA-DR PPI network, it can be found that the top 30 degree targets are INS, ALB, IL6, VEGFA, TNF, FN1, EGF, MMP9, EGFR, MAPK1, IGF1, CXCL8, SRC, STAT3, CASP3, MAPK8, IL1B, JUN, IL10, CCL2, FGF2, PTGS2, ICAM1, ESR1, NOS3, MMP2, TLR4, CXCL12, CRP, and SERPINE1. They may be the core target of tanshinone IIA in the treatment of DR. GO enrichment analysis shows that the main biological processes are mainly hypoxia, positive regulation of ERK1 and ERK2 cascade, steroid hormone-mediated signaling pathway, inflammatory response, angiogenesis, platelet degranulation, positive regulation of cell proliferation, and so on. In addition, the enrichment of the KEGG pathway shows that tanshinone IIA is mainly related to the VEGF signaling pathway, apoptosis, Fc epsilon RI signaling pathway, HIF-1 signaling pathway, PI3K-Akt signaling pathway, Ras signaling pathway, Rap1 signaling pathway, TNF signaling pathway, and so on. This suggests that tanshinone IIA may act on DR through related signaling pathways such as inflammation, angiogenesis, and immune factors.

VEGF is a highly specific mitogen of vascular endothelial cells, which can stimulate the mitosis and migration of vascular endothelial cells, increase the permeability of blood vessels, induce the formation of capillary cavities, and play an important role in neovascular retinopathy. Diseases such as retinal vein occlusion, proliferative diabetic retinopathy, and retinopathy of prematurity have similar pathogenesis [32]. That is, in the retinal hypoxia and ischemic environment, a variety of cells secrete VEGF, and its high expression can lead to retinal neovascularization [32]. In addition, VEGF can also cause retinal neovascularization by affecting the activity of other cytokines and pathways. It upregulates the expression level of ICAM-1 in retinal blood vessels, enhances platelet aggregation, promotes leukocyte adhesion, continues to aggravate retinal ischemia and hypoxia, and accelerates neovascularization [33]. Under a low-oxygen and high-glucose environment, VEGF overexpression can induce the activation of the downstream PI3K-AKT signaling pathway through receptors KDR and FIT-1. This can accelerate the migration and proliferation of endothelial cells, forming new microvascular cavities [34], and accelerate the development of the disease.

Protein kinase B (AKT) belongs to the serine/threonine protein kinase family, which controls important cell functions such as proliferation, apoptosis, metabolism, and transduction. It is closely related to the formation of neovascularization [35] and is regarded as an effective method for the treatment of pathological angiogenesis [36]. Studies have shown that the intravitreal injection of AKT inhibitors into the vitreous cavity of oxygen-induced retinopathy model mice under hypoxic environment can significantly reduce the formation of retinal neovascularization [37]. It is suggested that AKT activity will affect the generation and development of neovascular retinopathy. AKT is a direct target downstream of PI3K. The PI3K-AKT pathway is rich in genes in the KEGG pathway analysis, and it is closely related to the VEGF signaling pathway, mTOR signaling pathway, and other pathways. In the proliferative phase of proliferative vitreoretinopathy (PVR), activation of the PI3K-AKT signaling pathway and its downstream mTOR signaling pathway will cause the balance of cell proliferation and apoptosis in the eye to be imbalanced, leading to the formation of PVR [38]. The epidermal growth factor (EGF) is the strongest growth factor that promotes the proliferation of retina pigment epithelial cells (RPE). EGF and its receptor EGFR can induce the proliferation and migration of RPE cells and can cause changes in their morphology [39], thereby promoting the occurrence and development of PVR. The combination of EGF and EGFR can activate the MAPK signal pathway and the PI3K/AKT signal pathway, regulate the expression of EGF, VEGF, and other cytokines, and jointly participate in the proliferation and migration of RPE cells [40].

Current research shows that tanshinone IIA can significantly improve the hemodynamic and hemorheological indicators of CRVO patients and increase the blood supply to the affected eye [41]. Tanshinone IIA has a certain inhibitory effect on the proliferation of HRECs cultured in a high-glucose environment, which can significantly inhibit the mRNA and protein expression of VEGF and ICAM-1 of HRECs in a high-glucose environment and shows a dose-concentration-dependent relationship [42]. Tanshinone IIA can inhibit the expression of HIF-1 in rat retinal Müller cells under the condition of AGEs and enhance the expression of glutamine synthetase, thereby reducing the production of VEGF and the concentration of glutamate [43, 44]. Tanshinone IIA can also inhibit the expression of HIF-1*α* and VEGF in choroidal neovascularization (CNV) animal models and inhibit the experimental CNV produced by semiconductor laser photocoagulation [45]. Tanshinone IIA can inhibit the proliferation of RPE cells under hypoxic conditions, block cells in the G0/G1 phase, and promote cell apoptosis, which may be related to its inhibitory effect on the HIF-1*α*/VEGF signaling pathway [46]. Therefore, the key pharmacodynamic molecules based on tanshinone IIA will play an important role in the biological process of treating DR in the future.

### 3.3. General Condition of Rats

**Figure 9:**
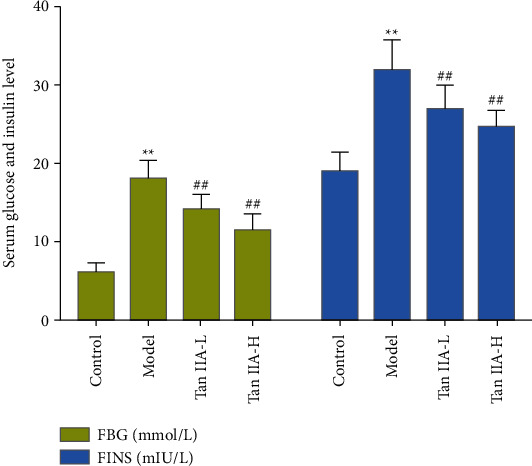
General condition of rats (Tan IIA-L: tanshinone IIA low-dose group; Tan IIA-H: tanshinone IIA high-dose group; ^*∗∗*^compared with the control group, *P* < 0.05; ^##^compared with the model group, *P* < 0.05).

After 2 months of tanshinone IIA treatment of DR rats, the changes in FBG and FINS were measured. Compared with the control group, the FBG and FINS of rats in the model group and the tanshinone IIA group decreased significantly increased (*P* < 0.05). Compared with the model group, the FBG and FINS levels decreased in the tanshinone IIA group (*P* < 0.05) (Figure 9).

### 3.4. Effect of Tanshinone IIA on Blood Lipids

**Figure 10:**
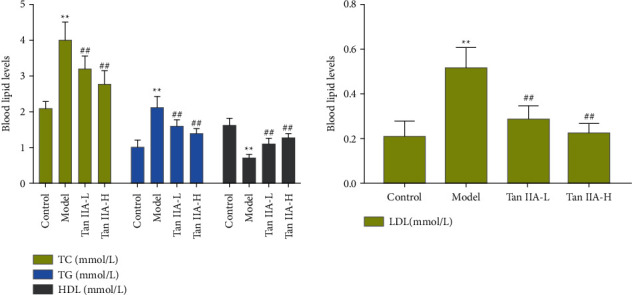
The effect of tanshinone IIA on blood lipids (Tan IIA-L: tanshinone IIA low-dose group; Tan IIA-H: tanshinone IIA high-dose group; ^*∗∗*^compared with the control group, *P* < 0.05; ^##^compared with the model group, *P* < 0.05).

The levels of TC, TG, and HDL-C in DR rats were higher than those in the control group, but after tanshinone IIA treatment, they were significantly reduced (*P* < 0.05). Compared with the control group, the model group and the tanshinone IIA group, HDL-C decreased significantly. Compared with the model group, the tanshinone IIA group had higher HDL-C levels (*P* < 0.05) (Figure 10).

### 3.5. Morphological Changes of Rat Retina

**Figure 11:**
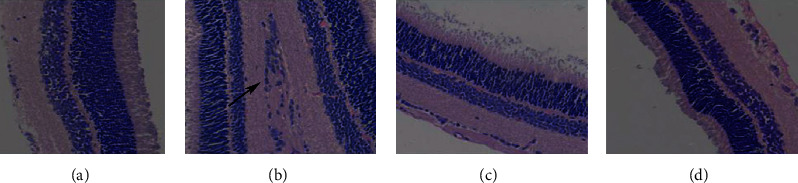
Morphological changes of rat retina (HE staining; 400x. The lesion is indicated by a black arrow). (a) Control. (b) Mode. (c) Tanshinone IIA-L. (d) Tanshinone IIA-H.

The tissue structure of the retina in the normal control group is clear, and the cells in the inner and outer nuclear layers are arranged regularly. In DR rats, the resolution of the retinal tissue layer is poor, the nerve fiber layer is edema, and the inner and outer nuclear layers are loosely arranged. The retinal cells of the tanshinone IIA low-dose group were basically layered, the cells were arranged well, and the inner and outer nuclear layers were slightly disordered; compared with the low-dose group, the tanshinone IIA high-dose group had further improved the retinal tissue structure in rats (Figure 11).

### 3.6. Effect of Tanshinone IIA on SOD, GSH-px, and MDA Levels in Rat Retina

**Figure 12:**
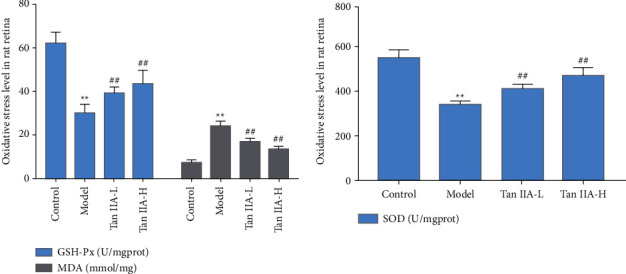
Effect of tanshinone IIA on SOD, GSH-px, and MDA levels in rat retina (Tan IIA-L: tanshinone IIA low-dose group; Tan IIA-H: tanshinone IIA high-dose group; ^*∗∗*^compared with the control group, *P* < 0.05; ^##^compared with the model group, *P* < 0.05).

The levels of SOD and GSH-px in the retina of diabetic rats were significantly reduced, and the level of MDA was significantly increased (*P* < 0.05). The SOD and GSH-px levels of the low-dose tanshinone IIA group and the high-dose tanshinone IIA group were significantly increased, and the MDA level was significantly decreased (*P* < 0.05) (Figure 12).

### 3.7. Effects of Tanshinone IIA on Serum TNF-*α*, IL-1*β,* and IL-6 Level

**Figure 13:**
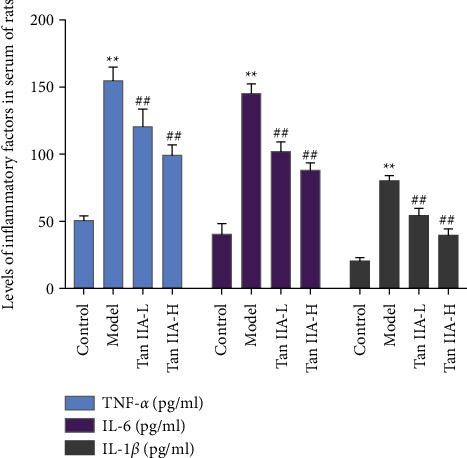
Effects of tanshinone IIA on the serum TNF-*α*, IL-1*β,* and IL-6 level (Tan IIA-L: tanshinone IIA low-dose group; Tan IIA-H: tanshinone IIA high-dose group; ^*∗∗*^compared with the control group, *P* < 0.05; ^##^compared with the model group, *P* < 0.05).

Compared with the control group, the serum levels of inflammatory cytokines IL-1*β*, IL-6, and TNF-*α* in the model group increased significantly (*P* < 0.05). Compared with the model group, the contents of IL-1*β*, IL-6, and TNF-*α* in the tanshinone IIA groups were significantly reduced (*P* < 0.05) (Figure 13).

### 3.8. Effect of Tanshinone IIA on the Expression of VEGF, IL-1*β*, IL-6, TNF-*α,* and Caspase-3 mRNA in Retinal Tissue

**Figure 14:**
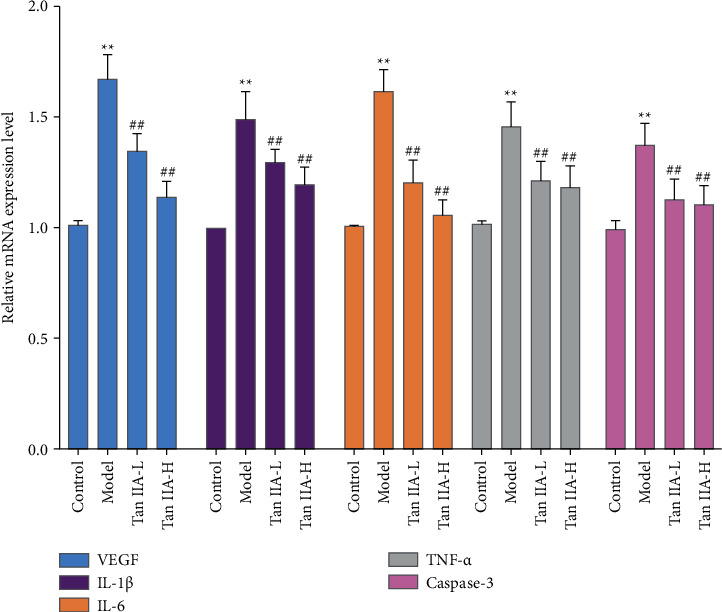
Effect of tanshinone IIA on the expression of VEGF, IL-1*β*, IL-6, TNF-*α,* and caspase-3 mRNA in retinal tissue (Tan IIA-L: tanshinone IIA low-dose group; Tan IIA-H: tanshinone IIA high-dose group; ^*∗∗*^compared with the control group, *P* < 0.05; ^##^compared with the model group, *P* < 0.05).

Compared with the normal group, the levels of VEGF, IL-1*β*, IL-6, TNF-*α*, and Caspase-3 mRNA in the retina of the model group were significantly increased (*P* < 0.05). Compared with the model group, the expression of VEGF, IL-1*β*, IL-6, TNF-*α*, and caspase-3 mRNA in the retina of the tanshinone IIA group was significantly reduced (*P* < 0.05) (Figure 14).

### 3.9. Effect of Tanshinone IIA on the Expression of Bcl-2, Bax, and VEGFA Protein in Retinal Tissue

**Figure 15:**
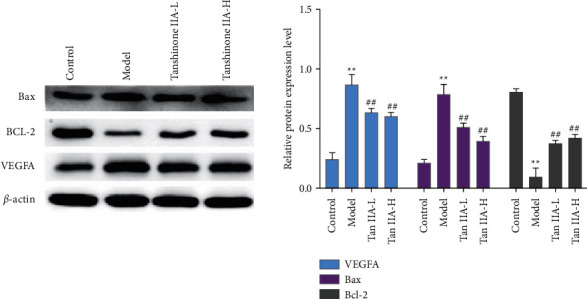
Effect of tanshinone IIA on the expression of Bcl-2, Bax, and VEGF protein in retinal tissue (Tan IIA-L: tanshinone IIA low-dose group; Tan IIA-H: tanshinone IIA high-dose group; ^*∗∗*^compared with the control group, *P* < 0.05; ^##^compared with the model group, *P* < 0.05).

The expression of Bcl-2 protein in retinal tissues of the model group was significantly decreased, and Bax and VEGFA were significantly increased (*P* < 0.05). Compared with the model group, the expression of Bcl-2 protein in the tanshinone IIA low-dose and high-dose group was significantly increased, and the expression of Bax and VEGFA protein in the tanshinone IIA high-dose group was significantly decreased (*P* < 0.05) (Figure 15).

Diabetic microvascular complications mainly include DR and diabetic nephropathy. According to whether the retina forms new blood vessels, it can be divided into nonproliferative diabetic retinopathy (NPDR) and proliferative diabetic retinopathy (PDR) [47]. NPDR has microvascular damage, such as telangiectasia and leakage, cotton wool spots, hard oozing, and other intraretinal microvascular abnormalities [48]. If it is not treated, it can evolve into a proliferative phase, forming neovascularization, vitreous hemorrhage, or anterior retinal hemorrhage, causing a severe loss of vision in patients and increasing the risk of blindness [49]. For the occurrence of DR, it is generally believed that hyperglycemia, insulin resistance, and genetic factors are the main pathogenic factors [50]. Excessive glucose transport changes the physiological effects of retinal cells, and glucose metabolism disorders cause capillary basement membrane thickening, endothelial cell proliferation, and damage to the self-regulation mechanism of retinal blood flow [51]. When hyperglycemia occurs, excessive free radicals are produced, and the antioxidant system is overloaded, which damages endothelial cells, causes vascular exudation, and stimulates neovascularization [51, 52]. The retina is composed of the retinal neuroepithelium and retinal pigment epithelium (RPE). RPE damage caused by any cause will have an irreversible effect on the visual function. The level of oxidative stress in the retinal tissue of diabetic patients increases, which affects the normal physiological functions of the retinal neuroepithelial and RPE layer, and the retina is highly sensitive to oxidative stress [53]. The excessive activation of inflammatory response and oxidative stress plays a very important role in the occurrence and development of DR in diabetic patients. Inhibition of retinal inflammation plays a protective role in diabetic kidney injury. Many inflammatory mediators such as IL-6, TNF-*α*, IL-1*β*, and ICAM-1 are increased in mice with diabetic complications, and they are involved in mediating the production of chemokines, the infiltration of inflammatory cells into the retina, and tissue damage [54]. Proinflammatory cytokines (such as TNF-*α*, IL-1*β,* and IL-6) and monocyte chemoattractant protein-1 (MCP-1) would chemoattract inflammatory cells to adhere to retinal capillaries (leukocyte stasis). The release of free radicals and proinflammatory cytokines leads to increased vascular permeability, breakdown of the blood-retinal barrier (BRB), and loss of capillary pericytes [55, 56]. Recent studies have also shown that proinflammatory mediators such as VEGF, NO, cytokines, chemokines, angiotensin II, renin-angiotensin system, eicosanoids, and lipids may lead to diabetic macular edema, ischemia, and new blood vessel formation [57]. Inhibition or blocking of proinflammatory molecules can prevent the development of DR in animal models [58–60]. In addition, in diabetes, the level of oxidative stress increases in the retina. SOD, GR, GPX, and CAT increased significantly in animal diabetes models [61] and diabetic patients [62, 63]. At the same time, the antioxidant capacity of diabetic patients is reduced, and the oxidizing environment generated by hyperglycemia significantly increases the risk of DR. Current studies have found that blocking the conduction of the oxidative stress pathway can attenuate the production of hyperglycemia-mediated oxidative stress and inflammatory factors, thereby protecting streptozotocin-nicotinamide-induced diabetic rats [64]. In different experimental models, inhibitors of oxidative stress can stimulate the activities of SOD, CAT, and GPx and increase GSH levels [65–67]. It can also reduce NOS activity, ROS levels, and nitrosative stress levels in the blood and retinal vascular endothelial cells [68]. Current studies have also shown that the unsteady increase in blood glucose and the overproduction of ROS trigger phenotypic changes in blood vessels, including retinal ischemia, hyperpermeability, and the formation of diabetic macular edema (DME) [69]. In addition, these factors and stimuli can also cause the release of VEGF. Studies have shown that the release of VEGF from retinal pigment epithelial cells plays a key role in the correct choroidal capillary development [70, 71]. Excessive production of VEGF can cause retinal neovascularization [72, 73]. The formation of new immature fragile blood vessels on the surface of the retina in the late stage can cause vitreous hemorrhage and even traction retinal detachment, which can lead to severe and irreversible vision loss [74].

In this experiment, the contents of TNF-*α*, IL-1*β*, and IL-6 in the serum of DR rats were analyzed, and the results showed that their contents all increased. The pathogenesis of microvascular disease in DR rats is mainly related to the production of high levels of free radicals in oxidative stress. Studies have shown that diabetes exhibits an increase in oxidative stress and inhibition of antioxidant enzyme activity. During the process of oxidative stress damage to the retinal tissue, a large amount of MDA is produced, and a large amount of antioxidant enzymes SOD and GSH-Px are also consumed [75]. This study confirmed that the intervention of tanshinone IIA in diabetic rats can increase the levels of SOD and GSH-Px and reduce the content of MDA, thereby reducing tissue oxidative damage under pathological conditions. Current studies have shown that in DR, Bax/Bcl-2, and P53 proteins play an important role in oxidative stress-mediated cell apoptosis. It is an important molecule that regulates the pathway of mitochondrial apoptosis and has potential antiapoptotic effects. The expression levels of Bax and P53 directly reflect the degree of apoptosis [76]. This study found that the Bcl-2 protein in the retinal tissue of the diabetic rats decreased, and the Bax and P53 proteins increased, indicating that streptozotocin can induce tissue apoptosis. After the intervention of tanshinone IIA, the expression of Bcl-2 increased, and the expression of Bax and P53 decreased, further confirming that tanshinone IIA can inhibit the apoptosis of retinal tissue induced by high glucose. In addition, this research did not observe the toxicity of tanshinone IIA (20 mg/kg), and the existing published studies did not report significant biological toxicity of high-dose tanshinone IIA [77–82].

In summary, this study promotes the exploration of the pharmacological mechanism of tanshinone IIA in DR. Although tanshinone IIA has been used to intervene in clinical coronary heart disease [83–85] and diabetic patients [86, 87], more clinical studies are needed to determine the efficacy and safety of tanshinone IIA in DR. At present, the drugs for diabetic retina mainly include blood lipid regulation, blood sugar lowering and blood pressure drugs, and anti-VEGF drugs [88, 89]. Although anti-VEGF drugs are currently widely used in clinical practice, due to their short half-life and short duration of action, repeated administration is required to maintain the drug dose. Its long-term efficacy and safety need to be verified by more authoritative clinical trials [90]. This study and previous studies show that tanshinone IIA, as a multi-targeted drug that interferes with angiogenesis, antioxidative stress, and inhibits inflammation, can be combined with other drugs in the future. The current research shows the efficacy and safety of the tanshinone IIA sodium sulfonate injection in humans. Therefore, it is more likely that the tanshinone IIA sodium sulfonate injection will be used in DR patients in the future.

## 4. Conclusion

In summary, the integrated pharmacology method was used in this study to study the molecular mechanism of tanshinone IIA in the treatment of DR. This study first analyzed the key pharmacodynamic molecular targets of tanshinone IIA in the treatment of DR, constructed a target interaction network of tanshinone IIA in the treatment of DR, and then verified it with an animal model. This study mainly elaborated the potential mechanism of tanshinone IIA in the treatment of DR from the aspects of anti-inflammatory, antioxidation, regulation of cell proliferation, apoptosis, and neovascularization and provided a reference for the study of the clinical application and mechanism of tanshinone IIA in the treatment of DR.

## Figures and Tables

**Table 1:** Primer.

	Forward primer	Reverse primer
VEGF	5′-GGGCTCAGGACCACATCATAA-3′	5′-GGGCTCAGGACCACATCATAA-3′
IL-1*β*	5′-TCCTCTGTGACTCGTGGGAT-3′	5′-TCAGACAGCACGAGGCATTT-3′
IL-6	5′-ACAAGTGGGAGGCTTAATTACACAT-3′	5′-TTGCCATTGCACAACTCTTTTC-3′
TNF-a	5′-CATCTTCTCAAAACTCGAGTGACAAAG-3′	5′-TGGGAGTAGATAAGGTACAGCCC-3′
IL-1*β*	5′-TCCTCTGTGACTCGTGGGAT-3′	5′-TCAGACAGCACGAGGCATTT-3′
Caspase-3	5′-ATGCTTACTCTACCGCACCCG-3′	5′-GGTTAACACGAGTAGGATGTGG-3′
*β*-Actin	5′-CAGCTATGTGGGGGACGAAG-3′	5′-TCCGTTAGCAAGGTCGGATG-3′

## Data Availability

The data used to support the findings of this study are included within the article and the supplementary information files.

## Supplementary Materials

Supplementary Materials Table S1: tanshinone IIA potential targets and DR genes. Table S2: enrichment analysis results.
