# Supplementary material for: Study on the Antioxidant Effect of Tanshinone IIA on Diabetic Retinopathy and Its Mechanism Based on Integrated Pharmacology
Source: Evid Based Complement Alternat Med. 2022 Nov 17;2022:9990937. doi: 10.1155/2022/9990937 (PMC9691304; doi:10.1155/2022/9990937)
Supplement: Supplementary Materials — Table S1: tanshinone IIA potential targets and DR genes. Table S2: enrichment analysis results. [file 9990937.f1.zip › 9990937.f1/Table S1.pdf]

Table S1 Tanshinone IIA Potential Targets and DR genes

Group	Target or Genes
Tanshinone IIA	ALB
	APOA2
	STS
	MAPK1
	MAPK10
	PIM1
	MMP3
	CA2
	BMP2
	BCHE
	CFB
	PPIA
	CASP7
	MAPK8
	KIF11
	AKR1C2
	MAPKAPK2
	TTR
	ESR1
	PDPK1
	AR
	GC
	CDK5R1
	METAP2
	RXRA
	RORA
	PDE4D
	PGR
	EGFR
	MAOB
	PDE4B
	SRC
	CCNA2
	BCAT2
	ESRRG
	MAPK14
	HSP90AA1
	PNMT
	ADAM17
	DPP4
	TYMS
	SHBG
	TGFBR1
	DHODH
	F2
	PPARD

MMP13
AKR1B1
DUSP6
CHEK1
SULT2A1
SEC14L2
PTPN11
ESR2
PPARG
HSD11B1
HPGDS
GSK3B
GSTP1
HSD17B1
PPP5C
NR3C2
GSTA1
AKR1C1
LTA4H
CDK2
CES1
WAS
FNTA
F10
NR1H4
ADH1C
HDAC8
SOD2
PRKACA
CDK6
MDM2
GSR
PTPN1
ADH5
EPHX2
SULT2B1
CYP2C9
MMP8
MET
THRB
BACE1
TTPA
PGF
CTNNA1
ADK
NR1H2
MIF
MTAP

RBP4
EPHB4
TRAPPC3
DAPK1
FABP6
LCK
SORD
CCNT1
KDR
JAK3
ITK
NOS3
FKBP1A
ESRRA
ERBB4
PPARA
HMGCR
FECH
NQO1
SULT1E1
AKR1C3
SERPINA1
ABO
FGFR2
FGFR1
HCK
PCK1
PDK2
RARB
HNF4G
FABP3
PDE5A
CYP2C8
FABP7
NQO2
IGF1
DPEP1
IL2
NR1I3
NR1I2
S100A9
NR3C1
NR1H3
LSS
RARA
MMP2
ZAP70
ITGAL

BLVRB
MTHFD1
REN
PRKCQ
MMP12
RARG
RXRB
MAP2K1
BIRC7
PPP1CC
VDR
SYK
TEK
CRABP2
DCK
PLA2G2A
HNMT
AKT2
THRA
XIAP
BRAF
BHMT
SETD7
MME
DHFR
TGM3
TPH1
HSP90AB1
CASP3
GRB2
ABL1
CTSB
CTSK
PYGL
PADI4
F7
TGFB2
ELANE
PIK3R1
F11
DTYMK
LCN2
PSAP
SDS
FDPS
FOLH1
NT5M
OAT

	KIT
	BCL2L1
	PCTP
	GSTA3
	GSTM2
	ENSG00000160200
	CASP1
	ACADM
	ADAM33
	CTSS
	GPI
	PROCR
	GSTT2B
	GSTM1
	TPSB2
	UCK2
	INSR
	GART
	FGG
	RFK
	CALM1
	CALM2
	CALM3
DR Genes	VEGFA
	ACE
	PON1
	EPO
	AKR1B1
	SERPINF1
	SOD2
	AGER
	ICAM1
	HFE
	ALB
	IGF1
	CCL2
	INS
	ANGPT2
	FGF2
	NOS3
	VCAM1
	IL1RN
	SERPINE1
	HGF
	PRKCB
	FLT1
	CTGF

CXCL12
MMP9
CXCL8
SST
CAPN5
KDR
MIR21
AOC3
SORD
TNF
TEK
PGF
TGFB2
TGFB1
AGT
PLXDC2
AGTR1
CHN2
BMP6
EDN1
MIR192
ADM
COLEC12
MTHFR
PPARG
MIR377
IL6
REN
MIAT
GFAP
VTN
SEMA3E
ENSG00000285441
TIMP1
FGF1
SPARC
IGFBP3
OPN4
VDR
TLR9
KCNH3
ITIH2
MALAT1
NAGLU
ITGA2
SOD1
TCF7L2
SELE

CRP
NAA15
ADIPOQ
PLXDC1
BDNF
HMGB1
NOS2
APOE
VWF
HIF1A
LTA
PVT1
CCNL1
IGSF21
LEKR1
KLHDC7A
HLA-DQB1
INSR
TLR4
IL17A
SELP
SLC2A1
ARHGAP22
CXCL10
NPY
ANGPT1
NPHS1
CNDP1
STK38L
MIR216A
MIR217
MEG8
SNHG6
SNHG18
IL10
TIMP3
IGFBP1
CYR61
MIR200B
TAB2
TCF4
KCNK1
CREB5
RBFOX1
MPRIP
VSTM2B
LINC00917
LOC339529

KRT18P34
LOC100506023
LOC729200
LOC101928236
ADRB3
MMP2
JUN
UCP2
APLN
THBS1
IFNG
NFKB1
APLNR
ANGPTL4
IL1R1
HLA-DRB1
HS6ST3
TIMP2
HP
IL1B
MAPK8
PLAT
IGF2
PF4
UCHL3
MYSM1
CNR1
TNFRSF11B
FNDC5
ROBO1
PON2
THBD
GHR
GSTM1
PTGS2
ITGB3
PPARGC1A
CYBB
LOX
GSTT1
NGF
ALDH2
SDHB
SLIT2
FGF21
ADAMTSL1
RBP4
F3

RHO
POSTN
NID1
AKT3
CTSD
PTX3
TTR
HMGCR
AKR1B10
STAT3
CD40LG
UTS2
HLA-DQA1
EGF
SIRT1
CCL5
BAX
ITGB2
HNF1A
CDH5
TJP1
PLG
IL1A
ELN
IFNA2
CXCL9
TF
IGF1R
ANG
PIK3CG
MIF
KITLG
ROBO4
NRP1
PTGDS
CCL4
PSMD9
ZNRF1
GORAB
API5
CCL3
PLVAP
PRDX1
SERPINI1
CFI
CLSTN1
ST3GAL4
AR

GJA1
CASP1
FN1
GH1
CA1
SETD2
CHI3L1
