# Supplementary material for: Study on the Antioxidant Effect of Tanshinone IIA on Diabetic Retinopathy and Its Mechanism Based on Integrated Pharmacology
Source: Evid Based Complement Alternat Med. 2022 Nov 17;2022:9990937. doi: 10.1155/2022/9990937 (PMC9691304; doi:10.1155/2022/9990937)
Supplement: Supplementary Materials — Table S1: tanshinone IIA potential targets and DR genes. Table S2: enrichment analysis results. [file 9990937.f1.zip › 9990937.f1/Table S2.pdf]

Table S2 Enrichment ana

Category	Term
	GO:0001666
	GO:0070374
	GO:0043401
	GO:0006954
	GO:0001525
	GO:0002576
	GO:0008284
	GO:0007568
	GO:0043066
	GO:0045429
	GO:0045766
	GO:0043406
	GO:0000187
BP	GO:0000165
	GO:0014068
	GO:0001938
	GO:0043410
	GO:0048661
	GO:0030198
	GO:0030593
	GO:2000352
	GO:0042593
	GO:0010595
	GO:0030168
	GO:0051092
	GO:0050729
	GO:0048010
	GO:0050728
	GO:0005615
	GO:0005576
	GO:0070062
	GO:0005829
	GO:0009986
	GO:0031093
	GO:0005578
	GO:0043235
	GO:0072562
	GO:0031012
	GO:0009897
	GO:0005886
CC	GO:0005764
	GO:0005654
	GO:0045121
	GO:0005737

	GO:0043025
	GO:0005901
	GO:0031234
	GO:0005925
	GO:0005739
	GO:0005911
	GO:0090575
	GO:0030426
	GO:0005788
	GO:0003707
	GO:0004879
	GO:0005125
	GO:0005102
	GO:0042802
	GO:0008083
	GO:0004713
	GO:0005515
	GO:0008144
	GO:0008201
	GO:0005178
	GO:0002020
**MF**	GO:0046934
	GO:0042803
	GO:0030235
	GO:0005496
	GO:0005088
	GO:0004714
	GO:0001948
	GO:0019899
	GO:0004252
	GO:0005179
	GO:0004672
	GO:0005158
	GO:0046982
	hsa04066
	hsa04151
	hsa04014
	hsa04015
	hsa04668
	hsa04068
	hsa04931
	hsa04620
	hsa04722
	hsa04064
	hsa04010
	hsa04920

	hsa04917
	hsa04012
**gnaling Pathw**	hsa04062
	hsa03320
	hsa04370
	hsa04060
	hsa04664
	hsa04610
	hsa04210
	hsa04024
	hsa04150
	hsa04630
	hsa04611
	hsa00590
	hsa00480
	hsa04350
	hsa04390

lysis Results

Description	Count
response to hypoxia	35
positive regulation of ERK1 and ERK2 cascade	35
steroid hormone mediated signaling pathway	23
inflammatory response	48
angiogenesis	37
platelet degranulation	27
positive regulation of cell proliferation	50
aging	30
negative regulation of apoptotic process	47
positive regulation of nitric oxide biosynthetic process	18
positive regulation of angiogenesis	25
positive regulation of MAP kinase activity	18
activation of MAPK activity	22
MAPK cascade	32
positive regulation of phosphatidylinositol 3-kinase signaling	18
positive regulation of endothelial cell proliferation	17
positive regulation of MAPK cascade	18
positive regulation of smooth muscle cell proliferation	16
extracellular matrix organization	24
neutrophil chemotaxis	15
negative regulation of endothelial cell apoptotic process	11
glucose homeostasis	17
positive regulation of endothelial cell migration	12
platelet activation	17
positive regulation of NF-kappaB transcription factor activity	18
positive regulation of inflammatory response	14
vascular endothelial growth factor receptor signaling pathway	13
negative regulation of inflammatory response	13
extracellular space	132
extracellular region	139
extracellular exosome	138
cytosol	143
cell surface	50
platelet alpha granule lumen	18
proteinaceous extracellular matrix	26
receptor complex	18
blood microparticle	19
extracellular matrix	26
external side of plasma membrane	21
plasma membrane	134
lysosome	18
nucleoplasm	92
membrane raft	17
cytoplasm	148

neuronal cell body	20
caveola	9
extrinsic component of cytoplasmic side of plasma membrane	9
focal adhesion	22
mitochondrion	50
cell-cell junction	13
RNA polymerase II transcription factor complex	6
growth cone	10
endoplasmic reticulum lumen	13
steroid hormone receptor activity	26
RNA polymerase II transcription factor activity, ligand-activated sequence-specific DNA binding	17
cytokine activity	28
receptor binding	38
identical protein binding	56
growth factor activity	26
protein tyrosine kinase activity	23
protein binding	268
drug binding	18
heparin binding	23
integrin binding	18
protease binding	17
phosphatidylinositol-4,5-bisphosphate 3-kinase activity	14
protein homodimerization activity	45
nitric-oxide synthase regulator activity	7
steroid binding	10
Ras guanyl-nucleotide exchange factor activity	17
transmembrane receptor protein tyrosine kinase activity	11
glycoprotein binding	13
enzyme binding	27
serine-type endopeptidase activity	23
hormone activity	14
protein kinase activity	27
insulin receptor binding	9
protein heterodimerization activity	30
HIF-1 signaling pathway	32
PI3K-Akt signaling pathway	58
Ras signaling pathway	43
Rap1 signaling pathway	41
TNF signaling pathway	29
FoxO signaling pathway	30
Insulin resistance	27
Toll-like receptor signaling pathway	25
Neurotrophin signaling pathway	24
NF-kappa B signaling pathway	20
MAPK signaling pathway	34
Adipocytokine signaling pathway	17

Prolactin signaling pathway	17
ErbB signaling pathway	18
Chemokine signaling pathway	26
PPAR signaling pathway	15
VEGF signaling pathway	14
Cytokine-cytokine receptor interaction	29
Fc epsilon RI signaling pathway	14
Complement and coagulation cascades	14
Apoptosis	12
cAMP signaling pathway	22
mTOR signaling pathway	11
Jak-STAT signaling pathway	18
Platelet activation	15
Arachidonic acid metabolism	9
Glutathione metabolism	7
TGF-beta signaling pathway	9
Hippo signaling pathway	13

%	PValue	Genes	Fold Enri	Bonferron	FDR
9.090909	1.38E-22	EPO, ADM	8.992044	4.77E-19	3.80E-19
9.090909	2.48E-22	NRP1, EP	8.837895	8.60E-19	3.80E-19
5.974026	5.36E-22	ESRRA, R	17.83084	1.85E-18	5.47E-19
12.46753	8.42E-22	CRP, CXC	5.596556	2.91E-18	6.44E-19
9.61039	9.54E-21	GPI, NRP1	7.331886	3.30E-17	5.84E-18
7.012987	2.16E-20	SERPINA	11.58365	7.47E-17	9.44E-18
12.98701	1.33E-19	RARG, FL	4.74136	4.61E-16	5.09E-17
7.792208	5.74E-18	EPO, ITG	8.03445	1.98E-14	1.83E-15
12.20779	8.83E-18	GSK3B, R	4.564627	3.05E-14	2.46E-15
4.675325	1.71E-17	EDN1, HS	18.49792	5.92E-14	4.03E-15
6.493506	7.30E-17	FLT1, CX	9.606407	3.84E-13	1.40E-14
4.675325	8.39E-15	EDN1, TG	13.48153	2.92E-11	1.22E-12
5.714286	2.58E-14	MAP2K1,	9.085686	8.91E-11	3.59E-12
8.311688	4.21E-14	PLVAP, F	5.397188	1.46E-10	5.37E-12
4.675325	4.98E-14	TGFB2, F	12.23709	1.72E-10	6.10E-12
4.415584	2.13E-12	NRP1, JU	10.88726	7.37E-09	2.17E-10
4.675325	2.47E-12	FLT1, INS	9.819883	8.53E-09	2.43E-10
4.155844	3.22E-12	JUN, EDN	11.78386	1.12E-08	3.08E-10
6.233766	1.05E-10	POSTN, V	5.410956	3.62E-07	9.14E-09
3.896104	1.87E-10	TGFB2, E	10.04306	6.48E-07	1.55E-08
2.857143	2.79E-10	IL10, ANC	17.36015	9.63E-07	2.13E-08
4.415584	9.23E-10	GPI, TCF7	7.437832	3.19E-06	6.14E-08
3.116883	4.40E-09	NRP1, ED	11.52769	1.52E-05	2.54E-07
4.415584	6.49E-09	SYK, VW	6.532357	2.24E-05	3.61E-07
4.675325	8.12E-09	TGFB1, P	5.98053	2.81E-05	4.37E-07
3.636364	8.15E-09	ITGA2, PI	8.474694	2.82E-05	4.37E-07
3.376623	6.67E-08	NRP1, HS	7.978655	2.31E-04	2.83E-06
3.376623	1.93E-07	HGF, SER	7.271686	6.67E-04	7.37E-06
34.28571	9.56E-54	IL1RN, SP	4.650705	3.47E-51	3.01E-51
36.1039	2.63E-50	SPARC, E	4.097334	9.55E-48	4.14E-48
35.84416	2.63E-23	PLVAP, II	2.329865	9.53E-21	2.76E-21
37.14286	5.76E-19	PNMT, H	2.047222	2.09E-16	4.54E-17
12.98701	4.20E-18	NRP1, PL	4.378075	1.52E-15	2.65E-16
4.675325	6.74E-16	TGFB2, T	15.53182	2.42E-13	3.54E-14
6.753247	5.18E-10	SERPINA	4.604167	1.88E-07	2.33E-08
4.675325	1.39E-09	NRP1, FL	6.726378	5.04E-07	5.46E-08
4.935065	3.39E-09	BCHE, TG	5.932292	1.23E-06	1.19E-07
6.753247	4.07E-09	SERPINE1	4.168637	1.48E-06	1.28E-07
5.454545	2.58E-08	CXCL9, A	4.678991	9.36E-06	7.39E-07
34.80519	3.31E-08	IL1RN, SP	1.543173	1.20E-05	8.70E-07
4.675325	6.24E-06	ACE, SRC	3.779867	0.002263	1.51E-04
23.8961	7.34E-06	GPI, SETI	1.568307	0.00266	1.64E-04
4.415584	7.79E-06	ANGPT1,	3.916464	0.002824	1.64E-04
38.44156	2.15E-05	IL1RN, SP	1.345047	0.007776	4.23E-04

5.194805	4.14E-05	NQO1, GS	3.013228	0.014901	7.66E-04
2.337662	6.27E-05	HCK, PLV	6.571154	0.02252	0.001098
2.337662	8.70E-05	ITK, ZAP7	6.28125	0.031089	0.00132
5.714286	8.73E-05	NRP1, MA	2.67029	0.0312	0.00132
12.98701	8.80E-05	GSK3B, S	1.782807	0.031437	0.00132
3.376623	2.89E-04	ITK, SLC2	3.586967	0.099761	0.004145
1.558442	5.84E-04	RXRA, VI	8.628788	0.191024	0.007996
2.597403	7.69E-04	GSK3B, N	4.091236	0.243525	0.009819
3.376623	7.79E-04	BCHE, SE	3.213325	0.246467	0.009819
6.753247	9.01E-27	RARG, TH	20.5173	7.54E-24	6.65E-24
4.415584	1.56E-17	ESRRA, N	20.86802	1.31E-14	5.77E-15
7.272727	3.11E-15	GPI, IL1RI	7.030402	2.60E-12	7.65E-13
9.87013	6.53E-15	ITK, SRC,	4.757116	5.48E-12	1.20E-12
14.54545	8.27E-15	SERPINA:	3.304007	6.87E-12	1.22E-12
6.753247	2.96E-14	GPI, FGF1	7.092399	2.48E-11	3.65E-12
5.974026	2.50E-13	MAP2K1,	7.64207	2.09E-10	2.63E-11
69.61039	3.27E-13	IL1RN, SP	1.348118	2.73E-10	3.01E-11
4.675325	8.14E-13	ACE, HSP	10.46631	6.81E-10	6.68E-11
5.974026	1.16E-11	NRP1, PO:	6.352471	9.67E-09	8.54E-10
4.675325	1.92E-10	VCAM1, S	7.575617	1.61E-07	1.29E-08
4.415584	9.24E-10	SERPINA:	7.438106	7.73E-07	5.58E-08
3.636364	9.83E-10	EGF, PTPI	9.978635	8.22E-07	5.58E-08
11.68831	2.91E-09	PLVAP, M	2.724109	2.43E-06	1.53E-07
1.818182	3.43E-09	HSP90AA	38.66721	2.86E-06	1.69E-07
2.597403	4.53E-09	ESRRA, A	16.36707	3.78E-06	2.09E-07
4.415584	6.49E-09	ANGPT1,	6.532597	5.43E-06	2.82E-07
2.857143	8.21E-09	FLT1, ERI	12.79216	6.86E-06	3.36E-07
3.376623	2.02E-08	HSP90AA	8.83822	1.69E-05	7.83E-07
7.012987	4.15E-08	THRB, SR	3.583062	3.47E-05	1.53E-06
5.974026	8.26E-08	F10, HGF,	3.985864	6.91E-05	2.90E-06
3.636364	1.86E-07	EDN1, EP	6.581653	1.55E-04	5.97E-06
7.012987	1.86E-07	GSK3B, S	3.323565	1.55E-04	5.97E-06
2.337662	3.13E-07	PTPN1, PI	12.82967	2.62E-04	9.64E-06
7.792208	8.40E-07	ITGB2, PI	2.851039	7.02E-04	2.39E-05
8.311688	2.07E-19	FLT1, EPC	7.542763	5.12E-17	1.28E-17
15.06494	3.84E-19	GSK3B, F	3.804176	9.48E-17	1.59E-17
11.16883	5.18E-16	FLT1, PIK	4.305383	1.37E-13	1.28E-14
10.64935	1.13E-15	FLT1, SRC	4.417904	2.74E-13	2.33E-14
7.532468	6.08E-15	PIK3R1, P	6.132901	1.51E-12	8.38E-14
7.792208	4.08E-13	SETD7, PI	5.066035	1.01E-10	4.21E-12
7.012987	5.06E-13	GSK3B, S	5.657072	1.25E-10	4.82E-12
6.493506	1.70E-11	CXCL9, C	5.336861	4.21E-09	1.32E-10
6.233766	1.57E-09	GSK3B, M	4.525658	3.89E-07	6.97E-09
5.194805	4.50E-09	VCAM1, C	5.201906	1.11E-06	1.92E-08
8.831169	1.39E-08	FGF1, FGI	3.040956	3.43E-06	5.22E-08
4.415584	3.88E-08	STAT3, A	5.495442	9.58E-06	1.30E-07

4.415584	4.81E-08	GSK3B, M	5.418041	1.19E-05	1.57E-07
4.675325	1.70E-07	GSK3B, M	4.681715	4.20E-05	4.68E-07
6.753247	4.91E-07	GSK3B, IT	3.163094	1.21E-04	1.22E-06
3.896104	9.07E-07	PDPK1, A	5.066035	2.24E-04	2.08E-06
3.636364	1.77E-06	MAP2K1,	5.193378	4.37E-04	3.99E-06
7.532468	2.44E-06	CXCL9, C	2.700495	6.02E-04	5.40E-06
3.636364	6.39E-06	MAP2K1,	4.658765	0.001578	1.32E-05
3.636364	7.57E-06	SERPINA1	4.591247	0.001868	1.54E-05
3.116883	6.86E-05	CASP7, A	4.379669	0.016807	1.20E-04
5.714286	1.53E-04	MAP2K1,	2.514254	0.037115	2.47E-04
2.857143	1.88E-04	PDPK1, PI	4.291572	0.045422	2.95E-04
4.675325	1.97E-04	IL10, EPO	2.809029	0.047574	3.06E-04
3.896104	0.001656	SYK, VWF	2.610956	0.335923	0.002095
2.337662	0.004969	CYP2C9, C	3.3386	0.707825	0.005813
1.818182	0.023629	GSTM2, G	3.105844	0.997278	0.023821
2.337662	0.030939	TGFB2, B	2.42446	0.999575	0.030939
3.376623	0.033946	GSK3B, T	1.948131	0.999803	0.033946
